# Hydroxytyrosol Inhibits MDSCs and Promotes M1 Macrophages in Mice With Orthotopic Pancreatic Tumor

**DOI:** 10.3389/fphar.2021.759172

**Published:** 2021-11-11

**Authors:** Botao Wang, Lei Yang, Tianyu Liu, Jing Xun, Yuzhen Zhuo, Lanqiu Zhang, Qi Zhang, Ximo Wang

**Affiliations:** ^1^ Graduate School, Tianjin Medical University, Tianjin, China; ^2^ Tianjin Key Laboratory of Acute Abdomen Disease Associated Organ Injury and ITCWM Repair, Institute of Acute Abdominal Diseases, Tianjin Nankai Hospital, Tianjin, China; ^3^ State Key Laboratory of Medicinal Chemical Biology, NanKai University, Tianjin, China; ^4^ Tianjin Key Laboratory of Acute Abdomen Disease Associated Organ Injury and ITCWM Repair, Institute of Integrative Medicine for Acute Abdominal Diseases, Integrated Chinese and Western Medicine Hospital, Tianjin University, Tianjin, China; ^5^ The Clinical Research Center of Tianjin for Treating Acute Abdominal Diseases with Integrated Medicine, Tianjin, China

**Keywords:** hydroxytyrosol, pancreatic cancer, MDSCs, M1 macrophages, stat3

## Abstract

The poor immunotherapy of pancreatic cancer is mainly due to its complex immunosuppressive microenvironment. The Mediterranean diet contributes to low cancer incidence. Hydroxytyrosol (HT) derived from olive oil has multiple health-promoting effects, but its therapeutic effect on pancreatic cancer remains controversial. Here, we evaluated the inhibitory effect of HT on mouse pancreatic cancer, and the effect of HT on the immune microenvironment. We found that HT can inhibit the proliferation of Panc 02 cells through signal transducer and activator of transcription (STAT) 3/Cyclin D1 signaling pathway. In the tumor-bearing mice treated with HT, the orthotopic pancreatic tumors were suppressed, accompanied by a decrease in the proportion of myeloid-derived suppressor cells (MDSCs) and an increase in the proportion of M1 macrophages. In addition, we found that HT inhibited the expression of immunosuppressive molecules in bone marrow (BM)-derived MDSCs, as well as down-regulated CCAAT/enhancer-binding protein beta (C/EBPβ) and phosphorylation of STAT3. Moreover, HT enhanced the anti-tumor effect of anti-CD47 antibody *in vivo*. HT combined with plumbagin (PLB) induced more Panc 02 cells death than HT or PLB alone. This combination therapy not only inhibited the accumulation of MDSCs, but also promoted the infiltration of CD4^+^ and CD8^+^ T cells in the tumors. In summary, HT is a potential immunomodulatory drug for the treatment of pancreatic cancer.

## Introduction

Many studies have shown that the Mediterranean diet can greatly reduce the incidence of local cardiovascular diseases and cancers, because it is rich in antioxidants and anti-inflammatory nutrients (Mentella et al., 2019). One of the ingredients is HT, an important phenolic compound found in olive oil (Robles-Almazan et al., 2018). Virgin olive oil intake contains 50–200 mg/kg of HT (de Pablos et al., 2019). More and more studies *in vivo* and *in vitro* have revealed the health-promoting effects of HT, such as anti-inflammatory, anti-oxidant, and anti-tumor. It can directly inhibit the generation of reactive oxygen species (ROS) to prevent oxidative stress damage to the DNA in normal cells and non-transformed cells, thereby avoiding the transformation of normal cells into tumor cells (Bernini et al., 2013). HT is found to inhibit lipopolysaccharide (LPS)-induced neuroinflammatory responses by the suppression of nuclear factor kappa-B (NF-κB) and extracellular regulated protein kinases (ERK) signaling pathway (Zhang et al., 2020). In terms of cancer research, high doses of HT has been found to induce apoptosis of thyroid cancer cells (Toteda et al., 2017). HT and colon metabolites of virgin olive oil can also induce cycle arrest and apoptosis of colon cancer cells (López de Las Hazas et al., 2017). Recently, HT has been found to increase p53 and γH2AX expression, reduce AKT expression, and remarkably reduce human melanoma cells viability (Costantini et al., 2020). These studies show that HT has an obvious direct inhibitory effect on tumor cells. But whether HT has a regulatory effect on tumor microenvironment (TME) is unknown, although it has anti-inflammatory effects.

Pancreatic cancer is one of the most aggressive malignant tumors, with a 5-years survival rate of <10% (Liot et al., 2021). Only 10–20% of patients are diagnosed with localized, surgically resectable pancreatic cancer (Strobel et al., 2019). It is predicted that pancreatic cancer will become the second leading cause of cancer-related deaths in the next 10 years (Neoptolemos et al., 2018). Unfortunately, immunotherapies approved by the FDA in other tumors have so far performed poorly on pancreatic tumors. The key reason is considered to be the immunosuppressive TME in pancreatic tumors (characterized by the typical poor infiltration of effector T cells and prominent myeloid cell inflammation) and the low mutation burden that produces few immunogenic antigens (Morrison et al., 2018). Therefore, improving the immunosuppressive TME of pancreatic cancer will become a new focus of drug development.

MDSCs are the main immunosuppressive cell subgroup in the TME of pancreatic tumors. This type of cell is one myeloid cell produced by the bone marrow under the continuous stimulation of relatively low intensity signals such as chronic infection, inflammation and tumor. Although similar in morphology and phenotype to neutrophils and monocytes, they have different genes expression, biochemical characteristics and functional activities (Gabrilovich, 2017). Their main role may be to protect the host from extensive tissue damage caused by an uncontrolled immune response related to inflammation or infection, but tumors can hijack and amplify this activity to protect themselves from the immune system (Bronte et al., 2016). MDSCs are implicated in the suppression of different immune cells, the main targets are T cells (Gabrilovich, 2017). Furthermore, they can enhance tumor cell stemness, promote angiogenesis, initiate formation of the pre-metastatic niche (Hinshaw and Shevde, 2019).

The differentiation and expansion of MDSCs depends on the inflammatory factors in the TME, including granulocyte-macrophage colony-stimulating factor (GM-CSF), granulocyte colony-stimulating factor (G-CSF), macrophage colony-stimulating factor (M-CSF), interleukin (IL)-6, IL-10, IL-1β(Groth et al., 2019). Accordingly, the accumulation and suppressive phenotypes of MDSCs are governed by a network of transcriptional regulators, which combine to prevent the differentiation of mature myeloid cells and promote the pathological activation of MDSCs(Condamine et al., 2015). The main factors include STAT3, interferon regulatory factor (IRF) 8, C/EBPβ, Notch, etc. (Gabrilovich, 2017). In mice, MDSCs are defined as CD11b^+^Gr-1^+^ cells, and there are two main subgroups: PMN-MDSCs (CD11b^+^Ly6G^+^Ly6Clo) and M-MDSCs (CD11b^+^Ly6G-Ly6Chi) (Bronte et al., 2016). These two different phenotypes of MDSCs can be differentiated and activated through IL-6, G-CSF, GM-CSF and other inflammatory factors to regulate STAT3 and C/EBPβ. The difference is that M-MDSCs mainly exert immunosuppressive effects by expressing inducible nitric oxide synthase (iNOS), Arginase 1 (ARG1), IL-10 and transforming growth factor beta (TGF-β), while PMN-MDSCs mainly exert immunosuppressive effects by expressing myeloperoxidase (MPO), ROS, ARG1 (Condamine et al., 2015; Gabrilovich, 2017). M-MDSCs have high plasticity and differentiation potential like monocytes. They can be further polarized into M1 and M2 macrophages in response to different microenvironments (Biswas et al., 2019; Chen et al., 2019). As supported by current knowledge, M1 macrophages exert tumoricidal activity, while M2 macrophages exert tumor-promoting effect. In addition, tumors can send a “don’t eat me” signal to macrophages through immune checkpoint CD47-SIRPα to prevent tumor cells from phagocytosis (Li et al., 2021). Therefore, the strategy to enhance immunotherapy should be to inhibit MDSCs and M2 macrophages, and promote the population of M1 and cytotoxic T lymphocytes.

In current study, we mainly explored the effect of HT on the immune microenvironment using mouse pancreatic cancer models, especially the regulatory effect of MDSCs. Our study found that HT can reduce the infiltration of MDSCs in the orthotopic pancreatic tumors. In mechanism, we revealed that HT inhibits the differentiation and function of mouse BM-derived MDSCs by inhibiting the expression of C/EBPβ and p-STAT3. Moreover, HT can promote the proportion of M1 macrophages in the tumors. Therefore, we further evaluated the effects of HT combined with anti-CD47 antibody on mouse pancreatic cancer progression, and our findings indicated that HT enhanced the anti-tumor effect of anti-CD47 antibody *in vivo*. PLB is a naphthoquinone with clear cytotoxicity against diverse cancer cells both *in vitro* and *in vivo*. Many efforts are currently using PLB as combination anticancer therapeutics (Tripathi et al., 2019). Here, we further evaluated the effects of HT combined with PLB on mouse pancreatic cancer progression and immune microenvironment. The results showed that this combination not only inhibited the infiltration of MDSCs, but also increased the proportion of M1 macrophages, CD4^+^ and CD8^+^ T cells in the tumors. These findings suggest that HT is a anti-tumor immunomodulatory drug, as well as propose new combination therapies can significantly improve immunosuppressive microenvironment of pancreatic cancer.

## Materials and Methods

### Cell Culture

Panc 02 cells (mouse pancreatic cancer cell line) were obtained from American Type Culture Collection. The cells were cultured in Dulbecco’s modified Eagle’s medium (DMEM) (Gibco, United States) supplemented with 100 μg/ml streptomycin and 100U/ml penicillin (Gibco, United States), and 10% fetal bovine serum (FBS) (Biological Industries, Israel) in a humidified atmosphere of 5% CO2 at 37°C.

### Apoptosis Assay

Panc 02 cells treated with different concentrations of HT (0, 50, 150, 200 μM) for 48 h or combined with 5 μM PLB for 24 h were collected and stained with Annexin V and propidium iodide (PI) according to manufacturer’s instructions (Tianjin Sungene Biotech, China). Subsequently, apoptotic cells or necrotic cells were detected by flow cytometry (ACEA, United States).

### Proliferation Assay

1×10^6^ Panc 02 cells were stained with 0.5 μM carboxyfluoroscein succinimidyl ester (CFSE) (Invitrogen, United States) according to manufacturer’s instructions. These cells were plated in a 12-well plate, 1 × 10^5^ cells/well. At the same time, different concentrations of HT (0, 50, 150, 200 μM) were added. After 48 h, the cells were measured by flow cytometry. The proliferation index (PI) of cells in different treatment groups was analyzed and calculated by ModFit LT (Verity Software House, United States).

### Mouse Orthotopic Pancreatic Tumor Model

Six- to 8-week-old female C57BL/6 mice were purchased from the Experimental Animal Center of Military Medical Sciences (Beijing, China) and maintained in the specific pathogen-free (SPF) conditions. Orthotopic models of pancreatic cancer were established as described previously (Xi et al., 2020). Briefly, a 1 cm longitudinal incision was made in the left upper abdomen of the anesthetized mice. The tail of pancreas was fully exposed through a gentle traction of the spleen. Subsequently, 50 μl of 5 × 10^5^ Panc 02 cells suspended in PBS were injected into the pancreas by using an insulin syringe. Finally, the peritoneum and skin were sutured with “4–0” suture. On the third day after the model was established, the tumor-bearing mice received solvent (0.9% normal saline), HT (200 mg/kg) (Shanghai Aladdin, China) or HT (200 mg/kg) combined with PLB (1 mg/kg) (J&K Scientific, China) treatment, and the mice were sacrificed after continuous intraperitoneal injection for 10 days. Anti-CD47 antibody or IgG2a (100 μg/mouse) (BioLegend, United States) was intraperitoneally injected twice a week, or combined with daily HT intraperitoneal injection treatment.

### Isolation of Tumor Infiltrating Mononuclear Cells and Spleen Cells

The tumors were cut into approximately 1mm^3^ pieces and digested with 0.05 mg/ml of type-IV collagenase (Sigma, United States), hyaluronidase (Sigma, United States) and DNase I (Sigma, United States) at 37°C for 60 min. Single cells were obtained by grinding through a 70 μm strainer. Subsequently, mononuclear cells were obtained by density gradient centrifugation using 40% percoll and 80% percoll (GE, United States). The spleens were ground into single cells through 40 μm strainer (FALCON, United States). Further wherein the red blood cells were lysed using erythrocyte lysis buffer to obtain other spleen cells.

### Isolation of BM Cells and Induction of MDSCs

The femurs and tibias of tumor-bearing mice or healthy C57BL/6 mice were separated and the two bone ends were cut off, and then PBS containing 100 μg/ml streptomycin and 100 U/ml penicillin was used to flush out the cells. The cells were depleted of red blood cells and collected for further study. BM-derived MDSCs were induced according to previously published study (Marigo et al., 2010). The isolated BM cells of healthy mice were plated into 12-well plate, supplemented with 40 ng/ml GM-CSF (PeproTech, United States) and 40 ng/ml IL-6 (PeproTech, United States), or 40 ng/ml GM-CSF alone. At the same time, these cells were treated with different concentrations of HT (0, 50, 100 μM) for 4 days. Then, all the cells were collected for further experiment.

### Flow Cytometry

Tumor mononuclear cells, spleen cells and BM cells were stained with the following antibodies: CD11b-FITC, Gr-1-APC/PE/PE-Cy7, F4/80-APC, MHC II-PE, Ly6G-PE, Ly6C-PE-Cy7, CD3-FITC, CD4-PE, CD8-APC. The stained cells were incubated for 30 min, washed once with PBS, and then detected by NovoCyte (ACEA, United States). All these antibodies were purchased from Tianjin Sungene Biotech. All data was analyzed through NovoExpress (ACEA, United States). The cell gate strategy was as follows: MDSC (CD11b^+^Gr-1^+^), PMN-MDSC (CD11b^+^Ly6G^+^Ly6C^−^), M-MDSC (CD11b^+^Ly6G-Ly6C^+^), M1 macrophage (CD11b^+^F4/80^+^MHC II^+^), CD4^+^ T cell (CD3^+^CD4^+^), CD8^+^ T cell (CD3^+^CD8^+^).

### Intracellular ROS Assay

BM-derived MDSCs were harvested and resuspended in 100 μL PBS. DCFH-DA, a fluorescent probe (Nanjing Jiancheng Bioengineering Institute, China) for ROS was added to the cell suspension, and the cells were incubated at 37°C for 30 min. The cells were then washed twice with PBS. The intracellular ROS producation was detected by flow cytometry.

### Quantitative Real-Time PCR

BM-derived MDSCs were added with TRIzol reagent (Invitrogen, United States) to extract total RNA according to the manufacturer’s instruction. RNA was quantified by NanoDrop 2000 (Thermo Fisher Scientific, United States). Next, RNA was reverse transcribed into cDNA using First-Strand cDNA Synthesis SuperMix (TransGen Biotech, China). DNA amplification was detected by ABI 7500 (Applied Biosystems, United States) using SYBR Green Master Mix (Promega, United States). GAPDH was used as an internal control. The relative expression of genes was calculated using 2^−ΔΔCT^. The primer sequences involved are as follows:

GAPDH-F ATG​GTG​AAG​GTC​GGT​GTG​AAC​G, GAPDH-R CGC​TCC​TGG​AAG​ATG​GTG​ATG​G, ARG1-F CTC​CAA​GCC​AAA​GTC​CTT​AGA​G, ARG1-R AGG​AGC​TGT​CAT​TAG​GGA​CAT​C, TGF-β-F GGA​CCG​CAA​CAA​CGC​CAT​CTA​T, TGF-β-R TTC​AGC​CAC​TGC​CGT​ACA​ACT​C, CEBPβ-F CAC​GAC​TTC​CTC​TCC​GAC​CTC​T, CEBPβ-R GCT​CAG​CTT​GTC​CAC​CGT​CTT​C.

#### Western Blot

Total protein from BM-derived MDSCs or Panc 02 cells treated with different concentrations of HT was extracted using RIPA lysis buffer (Millipore, United States) containing proteinase inhibitor and phosphatase inhibitor cocktails. 10–20 μg protein of each sample was electrophoresed by SDS-PAGE and transferred to PVDF membrane (Millipore, United States). The membranes were blocked in TBST containing 5% non-fat dried milk for 1h, then incubated with primary antibodies including p-STAT3 (Cell Signaling Technology, United States), STAT3 (Cell Signaling Technology, United States), C/EBPβ (Santa Cruz, United States), ARG1 (Abclonal, China), Cyclin D1 (Proteintech, United States), p-AKT (Cell Signaling Technology, United States), AKT (Cell Signaling Technology, United States), p-NF-kB (abcam, United Kingdom), NF-kB (abcam, United Kingdom), β-actin (Abclonal, China) according to the recommended concentration. After incubating overnight at 4°C, membranes were washed 3 times with TBST, and incubated with anti-rabbit or anti-mouse HRP-conjugated secondary antibody (Santa Cruz, United States) for 2 h at room temperature. Finally, band detection was performed using ECL reagents (Millipore, United States). The integrated density of the target proteins were analyzed through Image J (National Institutes of Health, United States).

### Hematoxylin and Eosin Staining

Mouse pancreatic tumor tissues were fixed with 4% paraformaldehyde, then dehydrated and made into paraffin blocks. 4 μm-thick tissue sections were stained with HE (Servicebio, China) according to the instruction. Staining observation and photographing were performed using Leica microscope (Germany).

### Immunohistochemistry and Analysis

IHC was conducted with reference to previous studies (Liao et al., 2019; Wu et al., 2020). Paraffin sections of mouse pancreatic tumors were subjected to a standard xylene and alcohol gradient for deparaffination, and then antigen retrieval was carried out through Citrate Antigen Retrieval solution (Solarbio, China). Streptavidin-Biotin Assay Kit (ZSGB-BIO, China) was used for endogenous peroxidase blockade and antibody incubation. Sections were incubated with primary antibodies, including anti-Ki67 antibody (Beyotime, China) and anti-Ly-6G/Ly-6C (Gr-1) antibody (BioLegend, United States). After overnight at 4°C, sections were incubated with Biotin-conjugated Goat Anti-Rat or Rabbit IgG (Proteintech, United States). Sections were subsequently coloured by using DAB kit (ZSGB-BIO, China) and stained with hematoxylin. The expression of Gr-1 and Ki67 was quantitatively analyzed using ImageJ Fiji software according to the previous protocol (Crowe and Yue, 2019).

### Statistical Analysis

All data are shown as mean ± SD. Statistical analysis was performed by GraphPad Prism 7, Student’s unpaired *t*-test was used for comparison between two groups, and one-way analysis of variance (ANOVA) was used for comparison between multiple groups. Tukey’s Honestly Significant Difference test was used for comparison between two groups in multiple groups following one-way ANOVA. *p* < 0.05 indicates that the difference is statistically significant.

## Results

### HT Inhibits the Proliferation of Pancreatic Cancer Cells and the Growth of Orthotopic Pancreatic Tumor

**FIGURE 1:**
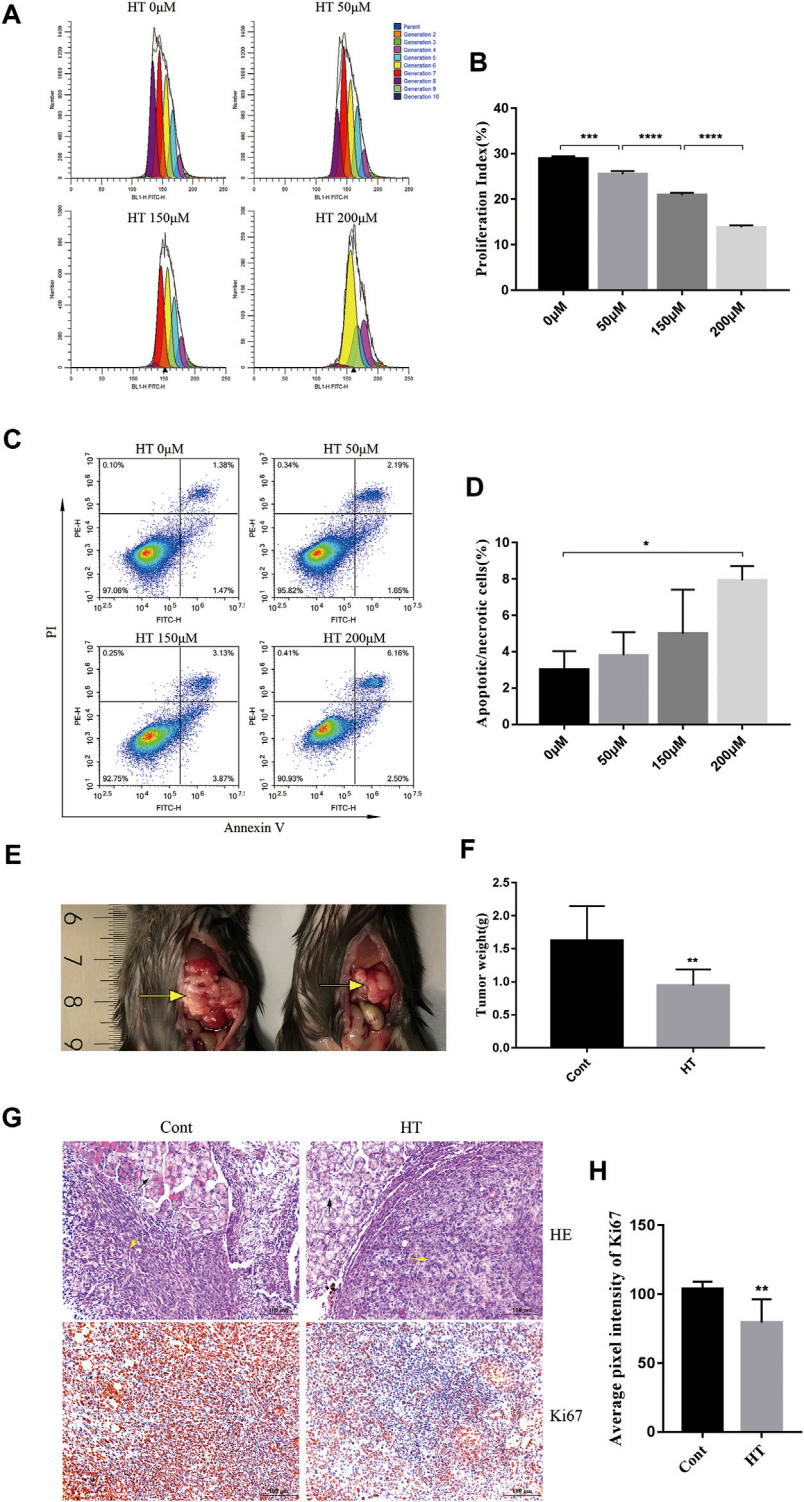
The inhibitory effect of HT on mouse pancreatic cancer *in vivo* and *in vitro*. The number of generations of Panc 02 cells have undergone after treatment with HT (0, 50, 150, 200 μM) for 48 h was detected using CFSE **(A)**. PI was further calculated and compared **(B)** (***p < 0.001, ****p < 0.0001). Panc 02 cells were treated with HT (0, 50, 150, 200 μM) for 48 h, apoptotic and necrotic cells (Annexin V + PI+/−) were detected by flow cytometry **(C,D)** (*p < 0.05, compared with 0 μM). The mice with orthotopic pancreatic tumor received HT (*n* = 9, 200 mg/kg) and normal saline (*n* = 9) intraperitoneal treatment for 10 days. Representative tumor *in vivo* **(E)**, and the tumor weight was compared **(F)** (**p < 0.01). Representative images of HE staining of mouse pancreatic tumor **(G)**, the black arrows are mouse pancrea tissue, and the yellow arrows are the tumor tissue. Ki67 expression in mouse pancreatic tumors was detected using IHC. Representative images and quantification of Ki67 from the control group and the HT treatment group **(G,H)**. Eight images from four mice in each group were analyzed for average pixel intensity and statistics **(H)** (**p < 0.01). All cell experiments were performed in three independent replicates.

We first explored the direct inhibitory effect of HT on mouse pancreatic cancer cells by proliferation and apoptosis assay. CFSE, a fluorescent dye is used to monitor the cell proliferation generation and calculate PI(Banks et al., 2011). By comparing the PI of cells treated with different concentrations of HT (0, 50, 150, 200 μM), we found that HT can significantly inhibit the proliferation of Panc 02 cells in a concentration-dependent manner (Figures 1A,B). After treating with different concentrations of HT (0, 50, 150, 200 μM), we also detected the apoptosis (Annexin V^+^PI^−^)/necrosis (Annexin V^+^PI^+^) of Panc 02 cells. Compared with 0 μM treatment, only the proportion of death (Annexin V+) cells in the 200 μM treatment group was significantly increased (Figures 1C,D). These results indicated that HT can inhibit the proliferation of pancreatic cancer cells, and high concentrations of HT can induce their death *in vitro*. We further evaluated the effect of HT on mouse pancreatic cancer *in vivo*. Orthotopic pancreatic tumors in mice treated with HT for 10 days were significantly suppressed (Figures 1E,F). Moreover, the expression of Ki67 in mouse orthotopic pancreatic tumors was significantly reduced after HT treatment (Figures 1G,H).

### HT Inhibits the Proliferation of Panc 02 Cells by Inhibiting p-STAT3/Cyclin D1 Signaling Pathway

**FIGURE 2:**
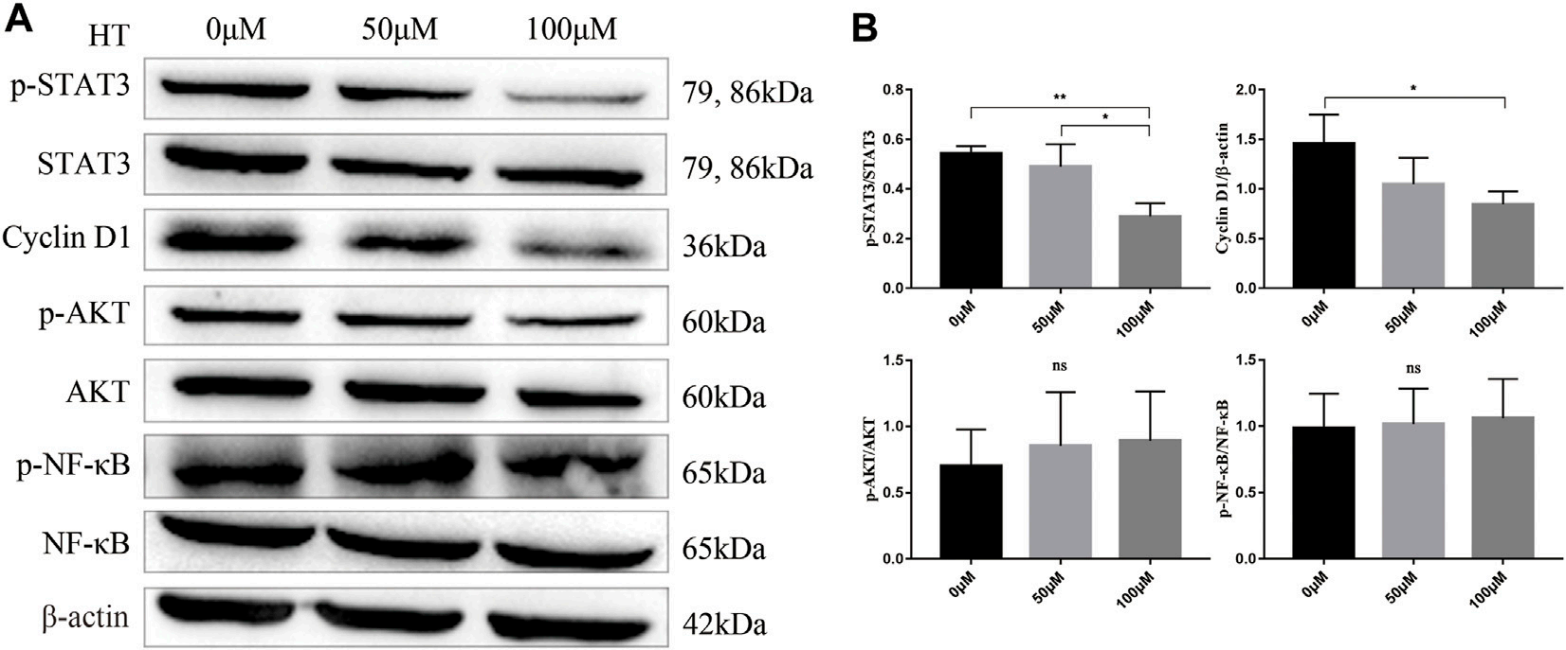
HT inhibits Panc 02 cells proliferation by inhibiting p-STAT3/Cyclin D1 signaling pathway. Panc 02 cells were treated with different concentrations of HT for 24 h, and the expression of p-STAT3, STAT3, Cyclin D1, p-AKT, AKT, p-NF-kB, NF-kB, β-actin in the cells was detected by western blot. Representative images and relative expression of proteins **(A,B)** (*p < 0.05, **p < 0.01). The analysis was based on three independent experiments.

Previous studies have shown that HT can inhibit the proliferation of a variety of tumor cells by inhibiting the activation of AKT and NF-kB (Zhao et al., 2014; Zubair et al., 2017). But our results showed that different concentrations of HT treatment for 24 h could not inhibit the phosphorylation of AKT and NF-kB in Panc 02 cells (Figures 2A,B). STAT3 is a central transcription factor in the progression of pancreatic cancer, which can promote the transcription of multiple genes encoding proliferation-related proteins, such as Cyclin D1 (Huang and Xie, 2012). Further exploration found that treatment with 100 μM HT can significantly inhibit the expression of p-STAT3 and Cyclin D1 in Panc 02 cells (Figures 2A,B).

### HT Reduces the Accumulation of MDSCs in Tumor-Bearing Mice

**FIGURE 3:**
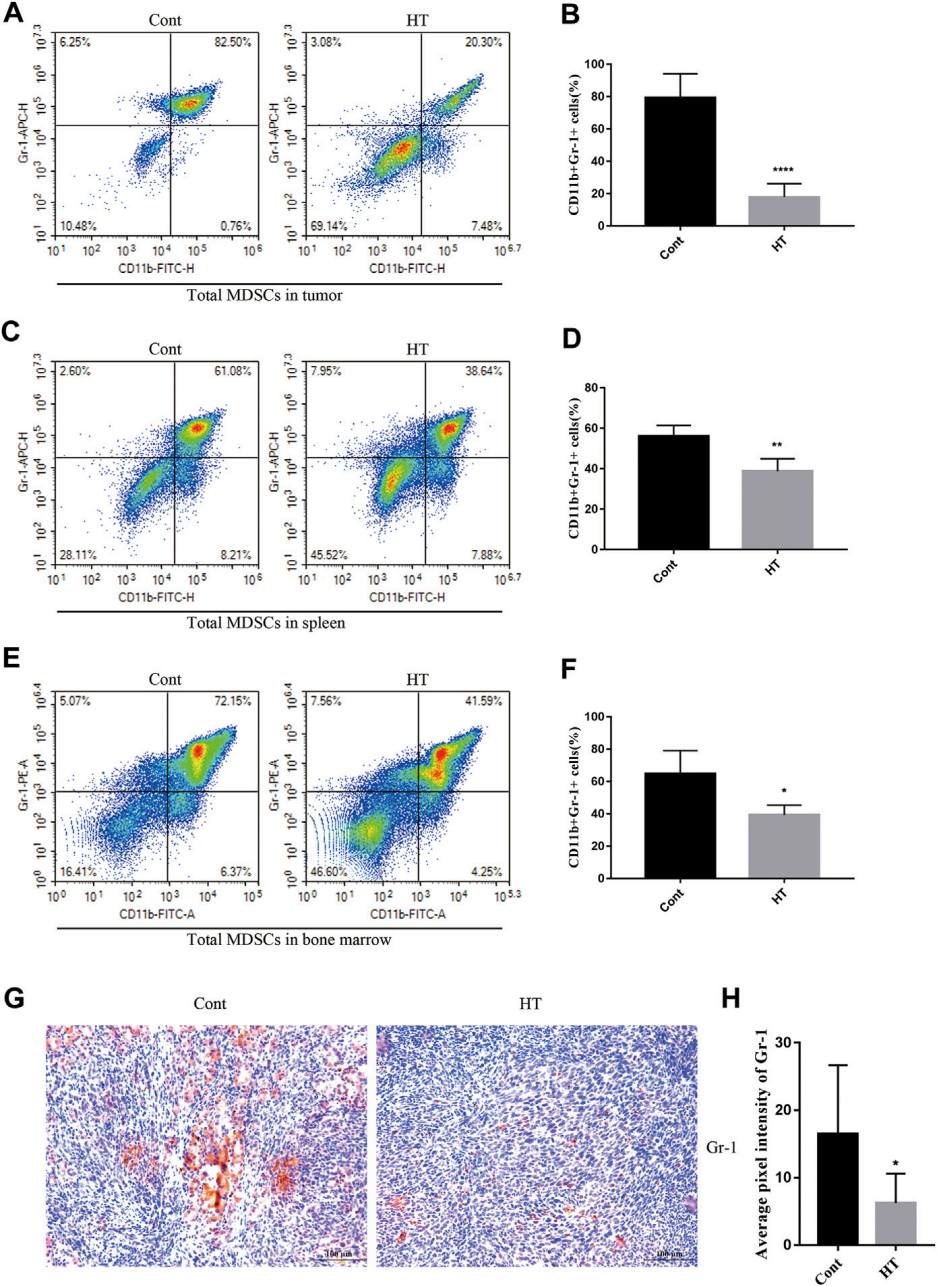
HT inhibits accumulation of MDSCs in the mice with orthotopic pancreatic tumor. The tumors, spleens and BM of mice in the control group and the HT treatment group were isolated. The proportion of CD11b + Gr-1+ cells in the tumors (*n* = 5 in each group) **(A,B)**, spleens (*n* = 5 in each group) **(C,D)** and BM (n = 3 in each group) **(E,F)** was detected using flow cytometry. Gr-1 expression in mouse pancreatic tumors was detected using IHC. Representative images and quantification of Gr-1 from the control group and the HT treatment group **(G,H)**. Eight images from four mice in each group were analyzed for average pixel intensity and statistics (*p < 0.05, **p < 0.01, ****p < 0.0001).

At present, there is no report on the regulation of tumor immunity by HT. MDSCs are the main immunosuppressive cells, which have been extensively studied and demonstrated to not only promote tumor progression, but also impair the anti-tumor immune responses elicited by therapeutic agents (Thyagarajan et al., 2019). We used flow cytometry to analyze the effect of HT on the proportion of MDSCs (CD11b^+^Gr-1^+^) in tumor tissues, spleens and BM of tumor-bearing mice. The results showed that HT can significantly reduce the accumulation of MDSCs in tumor tissues (Figures 3A,B), spleens (Figures 3C,D) and BM (Figures 3E,F). In addition, we analyzed the expression of Gr-1 in mouse pancreatic tumors through IHC, and found that the expression of Gr-1 in tumors from the HT treatment group was significantly reduced (Figures 3G,H). In order to further clarify the effects of HT on different subsets of MDSCs, we analyzed the proportion of PMN-MDSCs and M-MDSCs in the spleens. Compared with the control group, the proportions of PMN-MDSCs and M-MDSCs in the spleens of tumor-bearing mice were decreased after HT treatment (Supplementary Figures S1A,B).

### HT Inhibits the Differentiation and Function of BM-Derived MDSCs by Down-Regulating p-STAT3 and C/EBPβ

**FIGURE 4:**
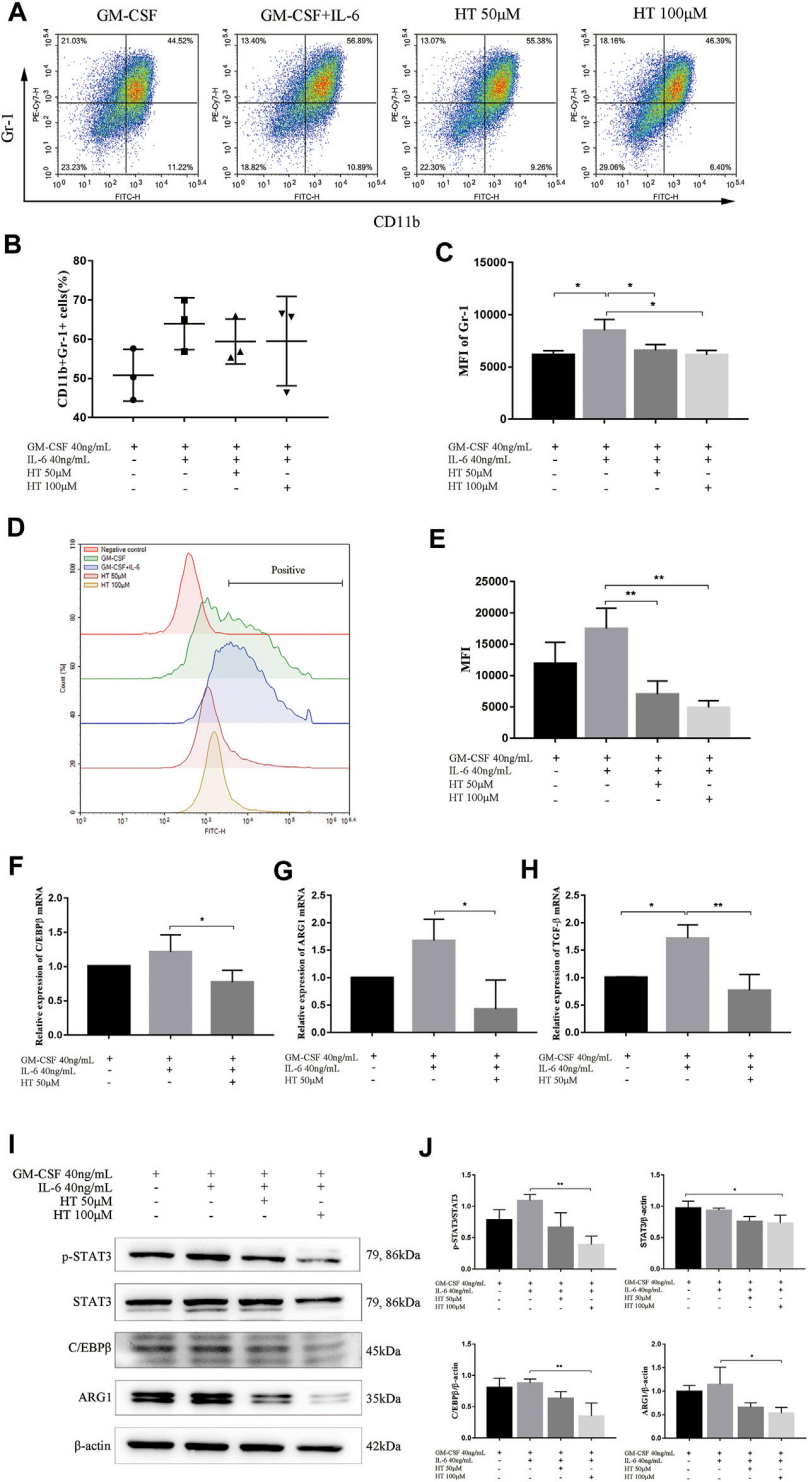
Effect of HT on the differentiation, function and signal pathway of BM-derived MDSCs. The BM cells of healthy C57BL/6 mice were isolated and supplemented with 40 ng/ml GM-CSF or/and 40 ng/ml IL-6 to induce differentiation of MDSCs. In addition, different concentrations of HT were added during this process. The proportion of MDSCs (CD11b + Gr-1+) and MFI of Gr-1 were measured by flow cytometry **(A–C)** (*p < 0.05). The ROS production of MDSCs in different treatment groups was measured using DCFH-DA **(D)**. Their MFI was analyzed and compared **(E)** (**p < 0.01). The transcription levels of C/EBPβ, ARG1 and TGF-β in MDSCs of different treatment groups were detected by qPCR **(F–H)** (*p < 0.05, **p < 0.01). After HT intervention, the expression of C/EBPβ, STAT3, and ARG1 in MDSCs was detected by western blot **(I)**. The integrated density of the target proteins was analyzed through Image J **(J)** (*p < 0.05). All analysis was based on three independent experiments.

We further explored the inhibitory effect of HT on BM-induced MDSCs *in vitro*. Previous study has shown that G-CSF, GM-CSF, and IL-6 allowed a rapid generation of MDSCs from precursors in BM of mouse and human. However, GM-CSF + IL-6 combination can produce enhanced expressions for CD11b and Gr-1 and more significant immunosuppressive activity (Marigo et al., 2010). In this study, we used this combination to induce MDSCs, while using GM-CSF alone as a reference to ensure the success of the induction. In the process of induction with 40 ng/ml GM-CSF and 40 ng/ml IL-6, 50 or 100 μM HT was added to detect the changes of CD11b^+^Gr-1^+^ cells population. The results showed that there was no statistical difference in the proportion of CD11b^+^Gr-1^+^ cells between the presence and absence of 50 or 100 μM HT (Figures 4A,B). However, compared with GM-CSF + IL-6 treatment, the mean flourscence indensity (MFI) of Gr-1 was significantly reduced after supplementation with 50 or 100 μM HT (Figure 4C).

MDSCs mainly perform immunosuppressive functions by expressing ARG1,TGF-β and generating ROS, and we then explored the effects of HT on them. We found that 50 and 100 μM HT can inhibit the production of ROS in BM-derived MDSCs (Figures 4D,E). At the same time, HT inhibited the transcription of ARG1 and TGF-β in BM-derived MDSCs, and further inhibited the protein expression of ARG1 (Figures 4G–J).

The phenotype and activity of BM-derived MDSCs depends on transcription factor C/EBPβ(Marigo et al., 2010). IL-6-mediated activation of STAT3 also sustains survival and accumulation of MDSCs(Smith et al., 2020). Our results showed that HT can inhibit the transcription of C/EBPβ, and inhibit the protein expression of C/EBPβ (Figures 4F,I,J). Meanwhile, HT inhibited the phosphorylation level of STAT3 (Figures 4I,J). The above results indicated that HT can inhibit the differentiation and function of BM-derived MDSCs by inhibiting the expression of C/EBPβ and p-STAT3.

### HT Promotes the Proportion of M1 Macrophages but not T Cells

The main inhibitory target of MDSCs in the TME is T lymphocytes. Our data revealed that HT can inhibit the accumulation of MDSCs and the expression of related immunosuppressive molecules in tumor-bearing mice. Therefore, we further analyzed the effect of HT treatment on the proportion of CD4^+^ T and CD8^+^ T cells. However, compared with the control group, HT treatment did not increase CD4^+^ T and CD8^+^ T infiltration in tumor tissues (Supplementary Figures S2A,B). Similarly, there was no difference in CD4^+^ T and CD8^+^ T cells of the spleens in the control group and the HT treatment group (Suppementary Figures S2C,D).

**FIGURE 5:**
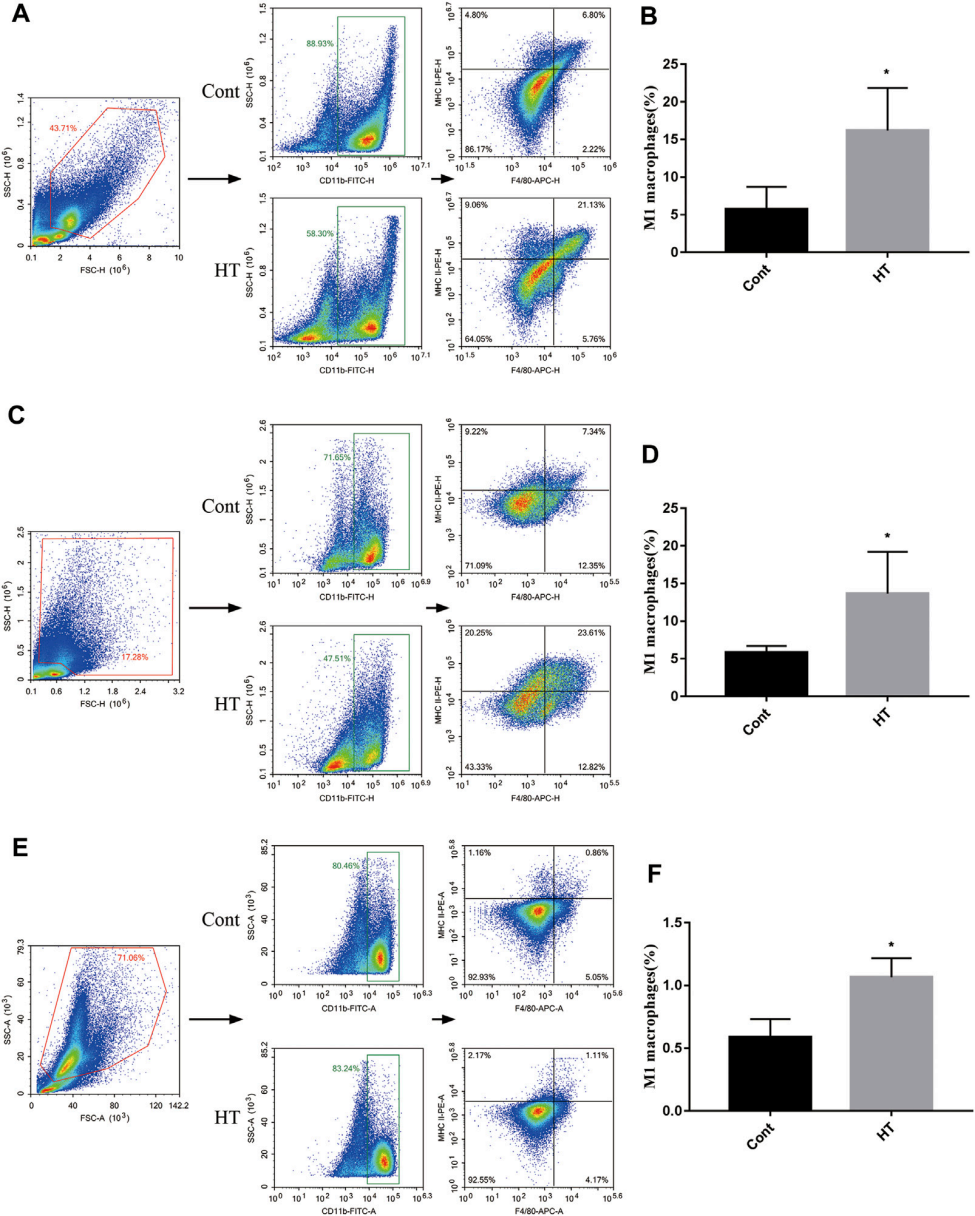
HT promotes the proportion of M1 macrophages in the mice with orthotopic pancreatic tumor. The tumors, spleens and BM of mice in the control group and the HT treatment group were isolated. The proportion of M1 macrophages (CD11b + F4/80 + MHCⅡ+ cells) in the tumors (*n* = 4 in each group) **(A,B)**, spleens (*n* = 4 in each group) **(C,D)** and BM (*n* = 3 in each group) **(E,F)** was detected using flow cytometry (*p < 0.05).

**FIGURE 6:**
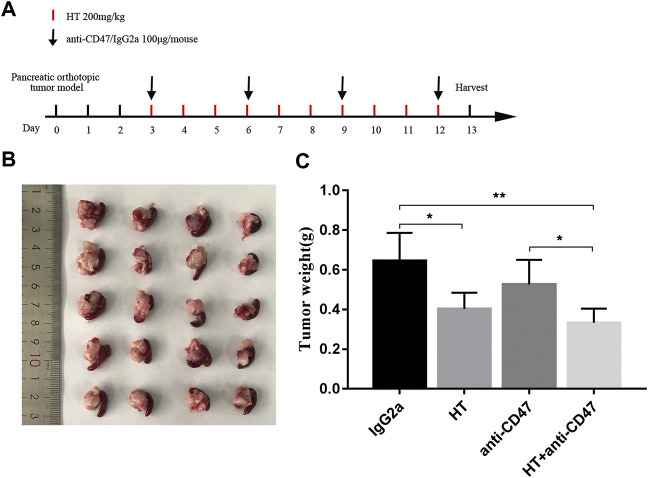
HT enhances the anti-tumor effect of anti-CD47 antibody. Tumor-bearing mice received the treatment schedule shown in **(A)**. The tumors after treatment in each group (*n* = 5) were separated and compared **(B,C)** (*p < 0.05, **p < 0.01).

In addition, we explored the effect of HT on M1 macrophages in tumor-bearing mice. We found that the level of CD11b^+^F4/80^+^MHC II^+^ cells was increased in the tumors (Figures 5A,B), spleens (Figures 5C,D), and BM (Figures 5E,F) of the tumor-bearing mice treated with HT. CD47 blocking can increase the efficiency of cancer cells clearance by macrophages. Therefore, we further evaluated the effects of HT combined with anti-CD47 antibody on mouse pancreatic cancer progression. We found that HT enhanced the anti-tumor effect of anti-CD47 antibody (Figures 6A–C). But compared with HT treatment, the combination of HT and anti-CD47 antibody did not further alleviate tumor weight.

### HT Combined With PLB Therapy Reshapes the Immune Microenvironment of Pancreatic Cancer in Mice

**FIGURE 7:**
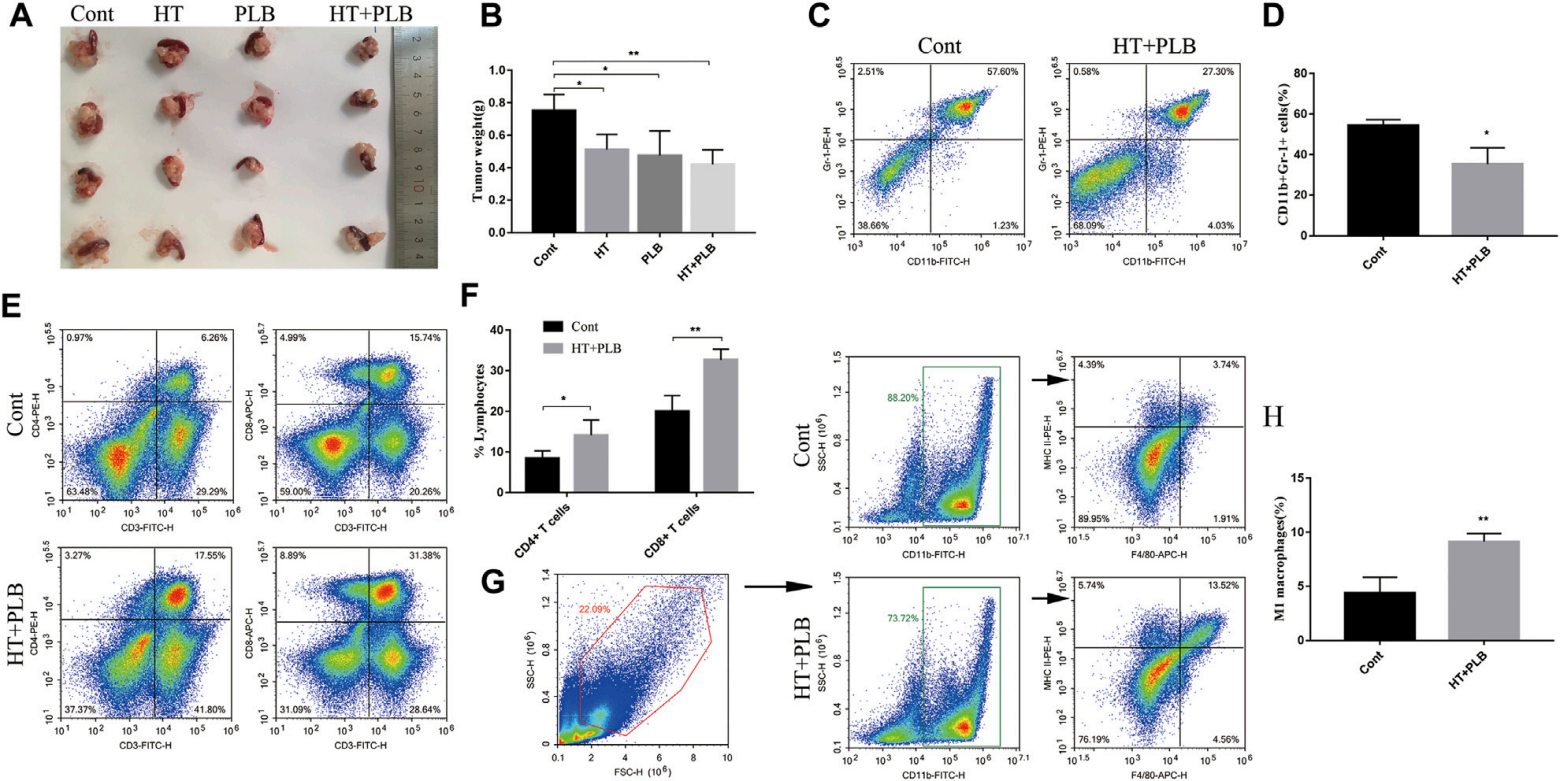
HT combined with PLB therapy reshapes the immune microenvironment of pancreatic cancer in mice. The mice with orthotopic pancreatic tumor received HT combined with PLB (HT 200 mg/kg, PLB 1 mg/kg) and normal saline intraperitoneal treatment for 10 days. Representative tumors *in vivo* **(A)**, and the tumor weight was compared (*n* = 4 in each group) **(B)** (*p < 0.05, **p < 0.01). Tumor tissues infiltrating-MDSCs (*n* = 3 in each group) **(C,D)**, CD4^+^ (*n* = 4 in each group) and CD8^+^ T cells (*n* = 3 in each group) **(E,F)**, M1 macrophages (*n* = 3 in each group) **(G,H)** in the control group and the combination therapy group were analyzed and compared (*p < 0.05, **p < 0.01).

To further enhance the anti-tumor effect of HT in pancreatic cancer, we chose PLB for combination therapy. *In vitro* studies showed that the combined treatment of 150 μM HT and 5 μM PLB resulted in higher Panc 02 cells apoptosis and necrosis than 150 μM HT or 5 μM PLB alone (Supplementary Figures S3A,B). We also used the mouse pancreatic orthotopic tumor model to evaluate the therapeutic effect. Compared with the control group, both HT and PLB treatments can suppress tumor progression, while HT combined with PLB treatment has more obvious difference (Figures 7A,B). However, there was no difference in tumor weight between HT or PLB treatment alone and combination treatment.

The previous results clarified the regulatory effects of HT on MDSCs. We have further evaluated whether the regulatory effect of HT on the immune microenvironment still exists in the presence of PLB. Compared with the control group, this combination therapy can also inhibit the accumulation of MDSCs (Figures 7C,D) and increase the proportion of M1 macrophages (Figures 7G,H) in the tumor tissues. This is consistent with the effect of HT treatment alone. Notablely, compared with the control group, the combined treatment of HT and PLB increased the infiltration of CD4^+^ T and CD8^+^ T cells (Figures 7E,F) in the tumor tissues of tumor-bearing mice. The above results indicated that the combined treatment of HT and PLB not only inhibits the growth of pancreatic tumors in mice, but also improves the immunosuppressive microenvironment and promotes anti-tumor immunity.

## Discussion

Tumors are not just composed of tumor cells. On the contrary, they are a complex “ecosystem” that includes all non-malignant host cells and non-cellular components, including but not limited to immune cells, endothelial cells, adipocytes, and stroma (Valkenburg et al., 2018). Tumor cells interact with the TME and often utilize factors that are conducive to tumor progression to make tumor cells immune escape. The pancreatic TME is characterized by a highly immunosuppressive microenvironment and dearth of high quality effector T cells (Mizrahi et al., 2020). The single treatment modality that induces effector T cell infiltration has not improved clinical outcomes (Lutz et al., 2011). Effective immunotherapy strategies may require a multifaceted approach, including induction of T cells infiltration, combined with immune stimulation methods, and targeting the immunosuppressive microenvironment (Mizrahi et al., 2020). MDSCs are a type of immunosuppressive cells that accumulate during tumor progression. In pancreatic cancer, the proportion of MDSCs was found to be significantly higher in patients’ peripheral blood and tumor tissues (Khaled et al., 2014). Markowitz J. et al. found that MDSCs in the peripheral blood of pancreatic adenocarcinoma patients with progressive disease were significantly increased, and the level of cytokines that promote MDSCs activation was also significantly increased in the plasma (Markowitz et al., 2015). These suggest that reducing the proportion of MDSCs or eliminating the function of MDSCs is beneficial to the treatment of pancreatic cancer. Therefore, the development of small molecule drugs targeting the immunosuppressive microenvironment is focused.

HT is a phenolic phytochemical with multiple pharmacological activities. It has been determined to be safe even at high doses and has no genotoxicity or mutagenicity *in vitro* (Echeverría et al., 2017). Moreover, the toxicological evaluation of HT based on rats did not find any toxicologically relevant effects, and proposed the dose of 500 mg/kg/d as the No Observed Adverse Effects Level (Auñon-Calles et al., 2013). Due to its bioavailability, biochemical characteristics, easy formulation and lack of toxicity, HT is regarded as an excellent food supplement by the nutraceutical and food industries (Bertelli et al., 2020). Previous study has shown that HT can selectively induce apoptosis in human pancreatic cancer cell MIA PaCa-2, but it has no effect on the viability of BxPC-3 and CFPAC-1 cells (0–300 µM) (Goldsmith et al., 2018). The effect of HT on mouse pancreatic cancer cell lines is still unclear, and there is still a lack of *in vivo* studies of HT in pancreatic cancer. In this study, we explored the direct inhibitory effect of HT on Panc 02 cells. On the other hand, we used a mouse orthotopic pancreatic tumor model to evaluate the anti-tumor effect of HT *in vivo*. Our results showed that HT can inhibit the proliferation of Panc 02 cells in a concentration-dependent manner (0–200 µM), and promote Panc 02 cells death at high concentration (200 µM). Mechanistically, we found that HT can inhibit Panc 02 cells proliferation by inhibiting p-STAT3/Cyclin D1 instead of inhibiting p-AKT/p-NFκb signaling pathway. Furthermore, HT can inhibit the growth of orthotopic pancreatic tumors in mice and reduce the expression of Ki67 in tumors. HT showed good therapeutic effects *in vivo* and *in vitro*.

Previous study has found that HT can inhibit the expression of inflammatory factors such as TNF-α, IL-1β and IL-6, and attenuate infiltration of activated immune cells in LPS-mediated sepsis in mice (Alblihed, 2021). HT can also inhibit mast cell degranulation induced by both immune and non-immune pathways (Persia et al., 2014). These studies suggest that HT may have ability to regulate immunity, but whether it has a regulatory effect on the tumor immune microenvironment has not been reported. Therefore, we further explored the impact of HT on the immune microenvironment of mouse orthotopic pancreatic tumor. The results showed that HT can inhibit the accumulation of MDSCs in tumor-bearing mice. In addition, HT can promote the proportion of M1 macrophages in the tumors, spleens and BM. We used BM-derived MDSCs to deeply explore the effect of HT on the differentiation of MDSCs and the expression of related immunosuppressive molecules. The results showed that HT can inhibit the differentiation of MDSCs, inhibit the expression of immune molecules (ARG1, TGF-β, ROS), and inhibit the expression of key transcription factors (C/EBPβ and p-STAT3) for the differentiation and function of MDSCs. This indicates that the treatment of HT in mouse pancreatic cancer is multi-approach. On the one hand, it has a direct inhibitory effect on tumor cells, and on the other hand, it has a regulatory effect on myeloid cells in tumor-bearing mice. In addition, based on the ability of HT to promote M1 macrophages, we further evaluated the anti-tumor effect of HT in combination with anti-CD47 antibody. The results indicated that HT can enhance the anti-tumor effect of anti-CD47 antibody. However, in this study, anti-CD47 antibody treatment alone did not achieve significant efficacy, and there was no difference between the antibody combined HT group and HT treatment group. The mechanism still needs further explanation.

PLB is a broad-spectrum anti-tumor drug that has been extensively studied. In this study, we used HT combined with PLB in order to obtain a better treatment effect. Although this combination therapy did not produce statistical differences with any of the single treatment in tumor weight. But surprisingly, this combination therapy reshaped the immune microenvironment of tumor-bearing mice, including inhibiting the accumulation of MDSCs, promoting the proportion of M1 macrophages, CD4^+^ T and CD8^+^ T cells. This combination should be further combined with other chemotherapeutic drugs and immune checkpoint antagonists, which is expected to have a new breakthrough in the treatment of pancreatic cancer.

## Conclusion

In summary, HT can inhibit mouse pancreatic cancer *in vivo* and *in vitro*. And our study demonstrated for the first time that HT inhibits the differentiation of MDSCs, the expression of ARG1, TGF-β and the generation of ROS in MDSCs by down-regulating the expression of C/EBPβ and p-STAT3. Moreover, HT can promote the proportion of M1 macrophages and enhance the anti-tumor effect of anti-CD47 antibody. We also innovatively proposed that HT combined with PLB can improve the immunosuppressive microenvironment of mouse pancreatic cancer, and it is expected to become potential drugs for immunotherapy. But there are still several limitations in the current study: First, the proportion of MDSCs in tumor-bearing mice decreased while the proportion of M1 macrophages increased after HT treatment. However, the source of M1 macrophages remains to be clarified. There is no direct evidence that MDSCs were polarized to M1 macrophages by HT. Second, although HT alone treatment obviously inhibited MDSCs, the proportion of T cells in tumor-bearing mice did not increase. This suggests that there may be other immunosuppressive factors that need to be further explored.

## Data Availability

The original contributions presented in the study are included in the article/Supplementary Material, further inquiries can be directed to the corresponding authors.
