# Supplementary material for: Hydroxytyrosol Inhibits MDSCs and Promotes M1 Macrophages in Mice With Orthotopic Pancreatic Tumor
Source: Front Pharmacol. 2021 Nov 11;12:759172. doi: 10.3389/fphar.2021.759172 (PMC8632498; doi:10.3389/fphar.2021.759172)
Supplement: Supplementary file 1 [file DataSheet1.docx]

Supplementary Material

## Supplementary Figures

##
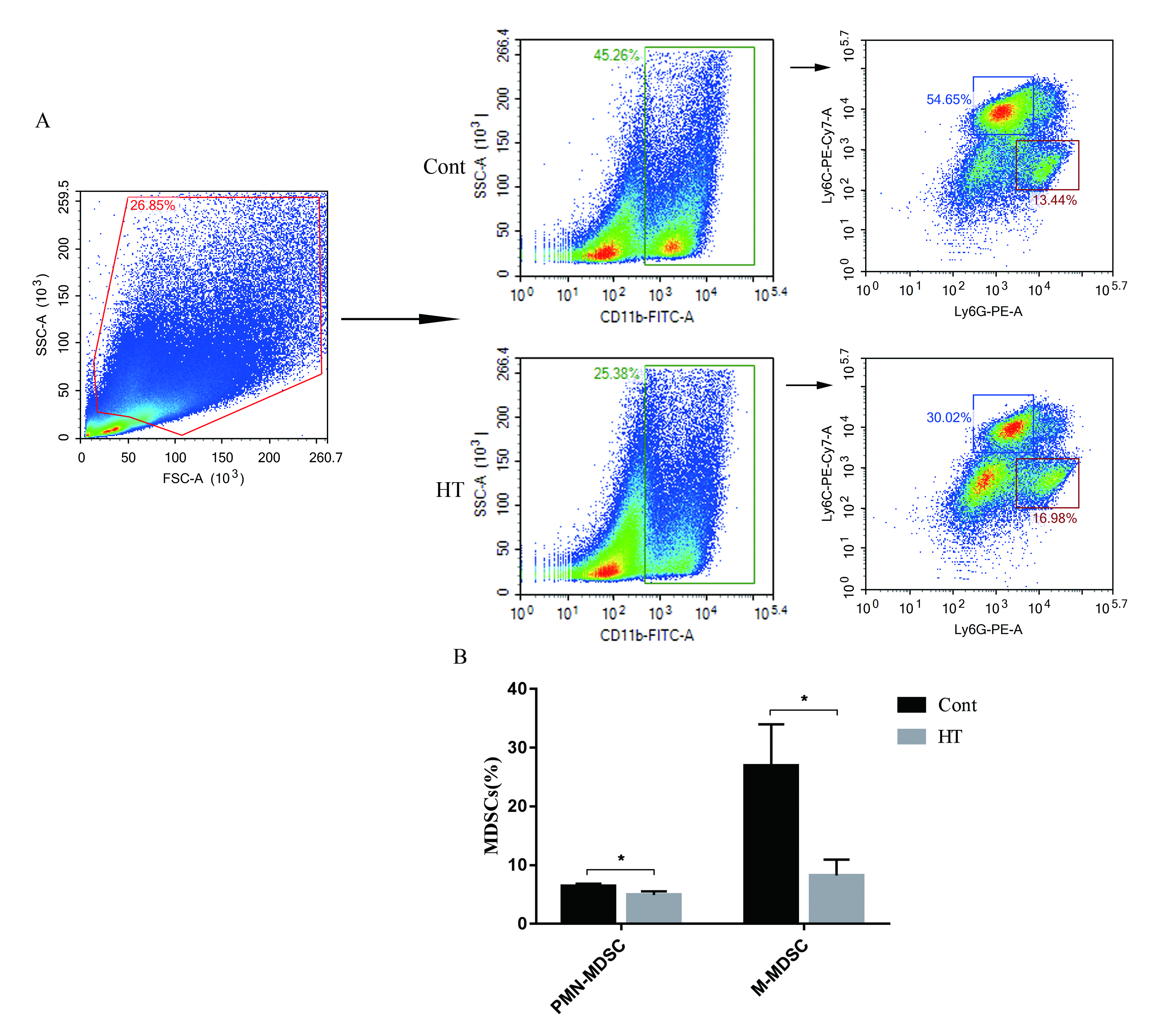
Supplementary Figure 1. HT inhibits the accumulation of different subtypes of MDSCs in spleens of tumor-bearing mice. The spleens of mice in the control group and the HT treatment group were isolated. Among them, PMN-MDSCs (CD11b+Ly6G+Ly6C-) (n=3 in each group) and M-MDSCs (CD11b+Ly6G-Ly6C+) (n=3 in each group) were further analyzed by flow cytometry (A,B) (*p<0.05).

**
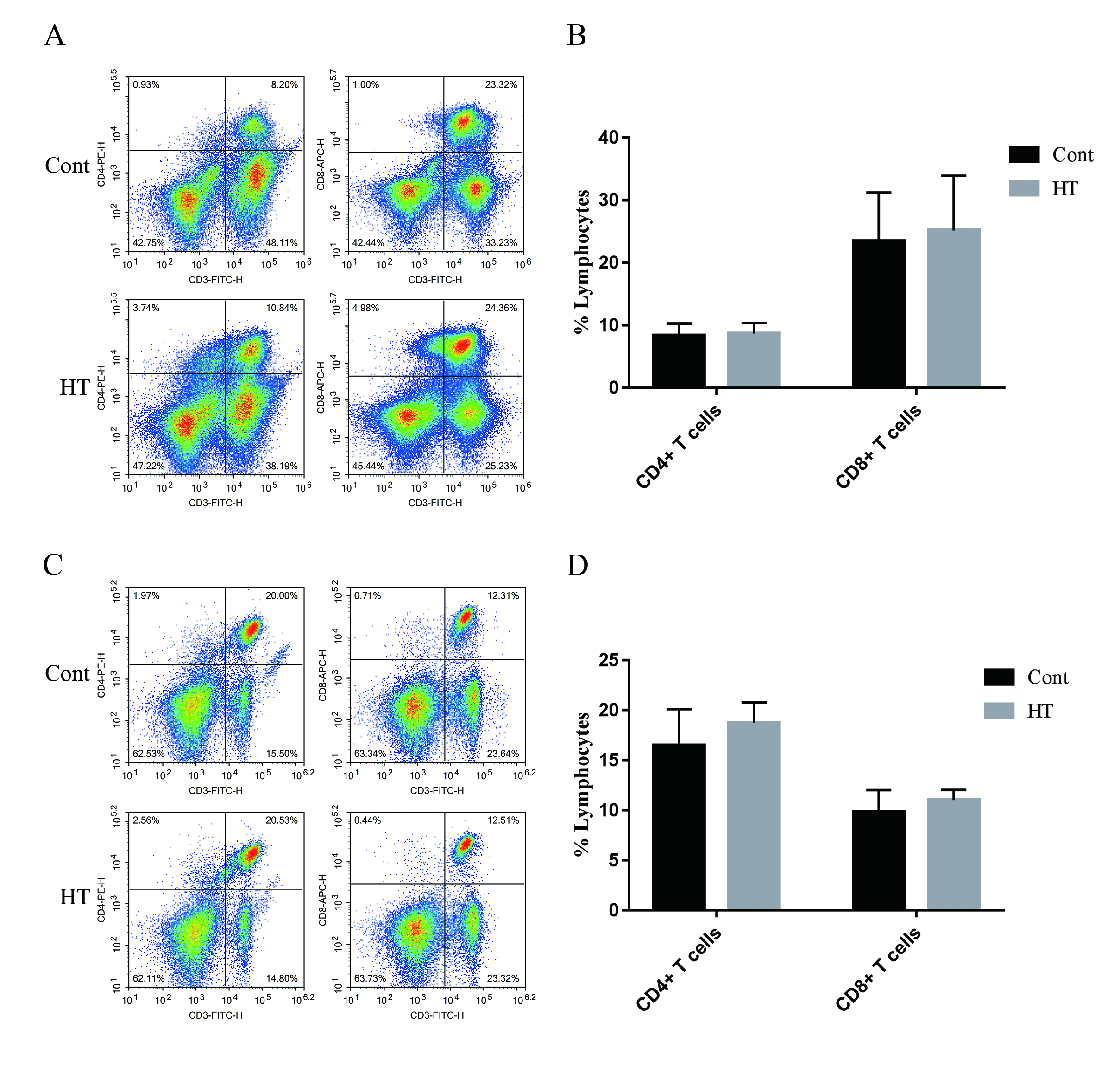
**

**Supplementary Figure 2.** HT cannot elevate T cells in the spleens and tumors of tumor-bearing mice. The spleens and tumors of mice in the control group and the HT treatment group were isolated. Among them, CD4+ T (CD3+CD4+) (n=4 in each group) and CD8+ T (CD3+CD8+) (n=4 in each group) were further analyzed by flow cytometry. There was no statistical difference between these two types of cells in the tumor (A,B) and the spleen (C,D).


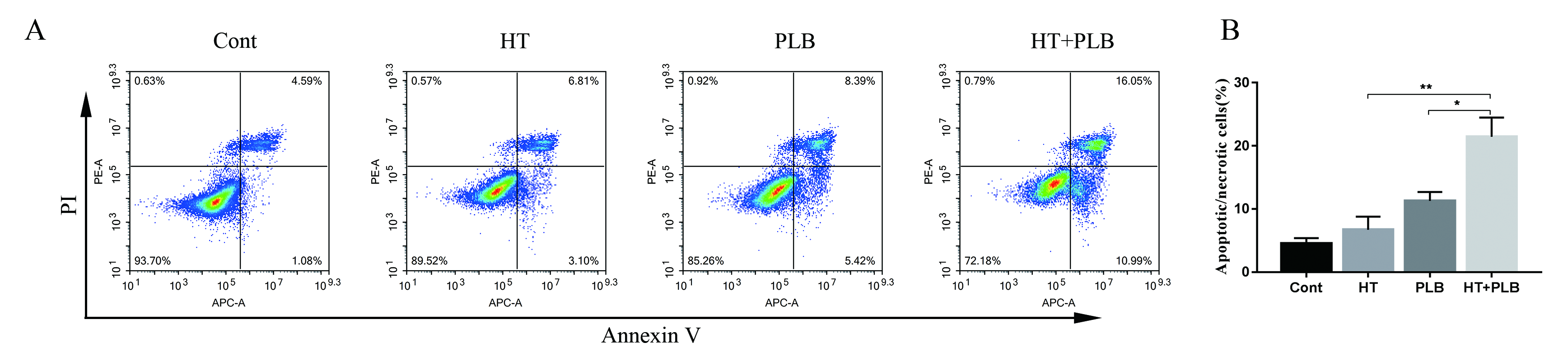


**Supplementary Figure 3.** HT combined with PLB enhances the ability to induce pancreatic cancer cell death. Panc 02 cells were treated with 150μM HT, 5μM PLB and a combination of the two for 24h, apoptotic and necrotic cells (Annexin V+PI+/-) were detected by flow cytometry (A,B) (*p<0.05, compared with PLB; **p<0.01, compared with HT). The analysis was based on three independent experiments.
